# Three-Dimensional Digital Image Correlation Based on Speckle Pattern Projection for Non-Invasive Vibrational Analysis

**DOI:** 10.3390/s22249766

**Published:** 2022-12-13

**Authors:** Alvaro Souto Janeiro, Antonio Fernández López, Marcos Chimeno Manguan, Pablo Pérez-Merino

**Affiliations:** 1 Department of Aeronautics, Universidad Politécnica de Madrid, 28040 Madrid, Spain; 2 Centre for Microsystems Technology, Ghent University and Imec, Technologiepark 126, 9052 Ghent, Belgium

**Keywords:** three-dimensional digital image correlation, speckle projection, full-field 3-D deformation, composite materials

## Abstract

Non-contact vibration measurements are relevant for non-invasively characterizing the mechanical behavior of structures. This paper presents a novel methodology for full-field vibrational analysis at high frequencies using the three-dimensional digital image correlation technique combined with the projection of a speckle pattern. The method includes stereo calibration and image processing routines for accurate three-dimensional data acquisition. Quantitative analysis allows the extraction of several deformation parameters, such as the cross-correlation coefficients, shape and intensity, as well as the out-of-plane displacement fields and mode shapes. The potential of the methodology is demonstrated on an Unmanned Aerial Vehicle wing made of composite material, followed by experimental validation with reference accelerometers. The results obtained with the projected three-dimensional digital image correlation show a percentage of error below 5% compared with the measures of accelerometers, achieving, therefore, high sensitivity to detect the dynamic modes in structures made of composite material.

## 1. Introduction

Non-contact optical sensing technologies combined with data processing algorithms play an essential role in non-invasively assessing a structure’s dynamic behavior. Structures with non-uniform material properties and/or complex geometry exhibit spatially local structural behaviors and are temporally transient. Therefore, full-field vibration-based measurements at very high spatial resolutions are required for the characterization and analysis of the structural dynamics for accurate identification of the modal parameters (i.e., modal shapes, frequencies and damping ratios) [1,2].

In recent years, optical-based vibration measurements have been proposed to measure structural dynamics, as they overcome the limitations of contact sensors (e.g., accelerometers), provide high-resolution measurements and do not change the structural dynamic behavior during testing. There exist a number of optical techniques for analyzing the dynamic response of a structure, such as laser Doppler vibrometry [3], electronic speckle interferometry [4,5], digital speckle shearography [6,7] and digital image correlation [8,9,10,11], among others. Laser Doppler vibrometry is the most extensively used technique for high spatial resolution vibration measurements owing to its superior resolution, sensitivity, robustness and non-contact nature, but it is an expensive and time-consuming method, which only offers pointwise measurements. Alternatively, scanning and continuous scanning laser Doppler vibrometry integrate a scanning system for full-field vibration measurements; however, different authors described (1) pseudo-vibration due to surface roughness and (2) measurement errors due to defocusing and motion artifacts at some scanning points [12,13].

Benefiting from the rapid development of high-speed advanced sensors and image processing techniques, digital image correlation (DIC) has been presented as a contactless alternative to the techniques above due to its simplicity, robustness, full-field, large deformation capabilities and accuracy of under 0.01-pixel precision with the use of the Newton–Rapshon method [14] and a bicubic interpolation [15,16]. DIC is a simple optical method that uses image registration to measure the strain and out-of-plane displacement of a structure and can be built up for full fields in three dimensions (3D) with the incorporation of two sensors or other strategies for stereo imaging (3D-DIC) [17,18,19,20].

For optimal use of 3D-DIC, it requires mechanical load synchronization with high-resolution and high-speed sensors to provide the tightest possible tolerance for spatio-temporal displacement calculation. Furthermore, the surface of interest must have a high-quality speckle pattern, which deforms together with the structure [21]. This configuration, together with the development of image processing algorithms, allows the matching of dense sets of points of the speckle pattern over a temporal sequence: before (unloaded), during (loaded) and after (unloaded) the mechanical excitation. On this matter, various authors have proposed different methodological approaches, on the one hand, to test the quality of the speckle pattern and, on the other hand, to improve the performance of the computational algorithms, such as the Shannon entropy [22], the mean intensity gradient [23], the mean intensity of the second derivative or, recently, the sum of the square of subset intensity gradients (SSSIG) [24,25].

The speckle pattern for 3D-DIC can be classified into three different groups: (i) a natural random pattern [26]; (ii) a synthetic speckle pattern: black dots randomly distributed on a white background by using a high-contrast paint sprayed with an airbrush air gun [27]; and (iii) a projected speckle pattern [28,29]. The synthetic speckle pattern is the most widely used for 3D-DIC applications; however, the physical surface treatment could be a source of contamination of the structure and manufacturing facilities, and it presents the main limitation of speckle distortion in high-temperature environments [30]. Moreover, it is a time-consuming method that does not ensure the desired size and density of the spots on the surface, and its invasive nature represents a clear limitation to perform measurements in optical materials or to apply it for in vivo measurements in biomedical applications [31,32]. However, the projection of the speckle pattern has multiple advantages with respect to artificial pattern speckles, i.e., the speckle pattern can be easily adapted to the experiment’s needs (the speckle pattern can be changed during the trial to achieve higher performance), and the speckle pattern is not painted on the surface of analysis, it is projected. This issue expands the technique to areas or experiments in which it is not possible to paint the speckle into the surface of a structure: optical surfaces (such as reflecting mirrors and lenses, solar cells and glass structures [33]) or biomedical applications (e.g., three-dimensional analysis of corneal biomechanics and intraocular pressure [34]). Moreover, it should be mentioned that vibration analysis fits very well with the speckle pattern projection in 3D-DIC since the displacement of the vibration modes always occurs out-of-plane [35,36,37,38].

In the last few years, two main directions have been described in the state-of-the-art for the development of non-invasive projected patterns: fringe patterns and laser speckle patterns. Fringe patterns have been combined with speckle patterns and a single camera for DIC in two dimensions and mechanical trials that required a low-frequency ratio of images [39,40]. The random granular effect in the laser speckle pattern has its origin in the high coherence of the light source that is projected to the sample and in the decorrelation when the sample moves in the out-of-plane direction. However, this technique entailed some increase in the uncertainty of the out-of-plane displacements [41]. In addition to the speckle pattern, different studies described other factors that affect the performance and accuracy of DIC: the criterion to evaluate the similarity between subsets [42,43], the iterative algorithm to process the information [44], the sub-pixel interpolation scheme [45,46], the quality and resolution of images [47], the speckle pattern quality [48] and the subset size [24].

This study proposes a custom-developed 3D-DIC methodology based on speckle pattern projection and image processing routines for full-field vibrational analysis at high frequency. The potential of the technique has been demonstrated on an Unmanned Aerial Vehicle (UAV) wing made of composite material, followed by experimental validation with reference accelerometers. The vibrational parameters, such as 3D displacement fields, natural frequencies and modal shapes, have been obtained and studied to demonstrate the capability of the developed methodology.

## 2. Materials and Methods

### 2.1. Projected 3D-DIC: Experimental Set-Up

**Figure 1:**
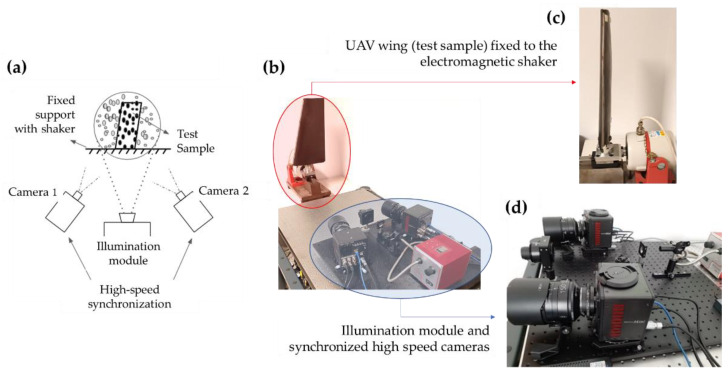
Experimental custom 3D-DIC set-up based on speckle pattern projection for full-field vibration measurements: (**a**) schematic layout of the experimental set-up and testing conditions (UAV wing made of composite material (test sample) clamped to the shaker); (**b**) set-up employed in the vibration test; (**c**) image of the UAV wing fixed to the electromagnetic shaker (lateral excitation); (**d**) optical channel for speckle pattern projection and high-speed sensors (distance of the tested sample during the measurements: 3.75 m; instantaneous field of view of the sensor: 0.5 mm).

Figure 1 illustrates the custom experimental set-up. Basically, the system comprises an optical channel for speckle pattern projection and two high-speed sensors. The optical channel consists of an on-axis illumination module (Broadband halogen fiber optic illuminator, OSL2, Thorlabs, Bergkirchen, Germany), which emits a stable power in the spectral range between 185 and 2000 nm, the optical elements required to collimate the beam and generate a uniform irradiance across the sample include two converging lenses and a printed speckle pattern in acetate. The off-axis imaging channel consists of two high-speed and high-resolution cameras (Mini AX100, Photron, Bucks, UK; 1024 × 1024 pixels at 4000 fps; pixel size: 20 µm; accuracy: 0.01 pixel) provided with a 150 mm focal length objective lens (Irix 150 mm f/2.8, Irixlens, Switzerland) and mounted on the X–Y-axis translation stage with a high precision rotation of 360 degrees (PR01, Thorlabs, Bergkirchen, Germany).

### 2.2. Projected 3D-DIC: Fundamentals

**Figure 2:**
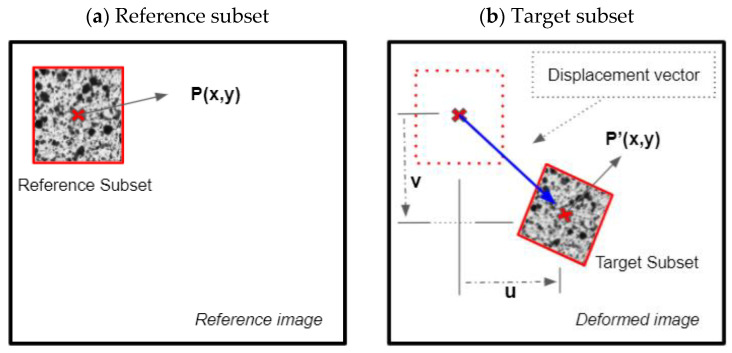
Schematic representation of DIC based on the correlation between the reference subset (**a**) and the target subset (**b**). The variables u and v represent the displacement experienced by the subset in the deformed image with respect to the reference image.

For high-frequency vibration analysis in projected 3D-DIC, the grey intensity of the reference image (unloaded state) is compared with the high-speed acquired images under deformation (loaded state). However, DIC is not directly applied between the reference and the deformed images; the acquired images are processed and divided into subsets (Figure 2).

**Figure 3:**
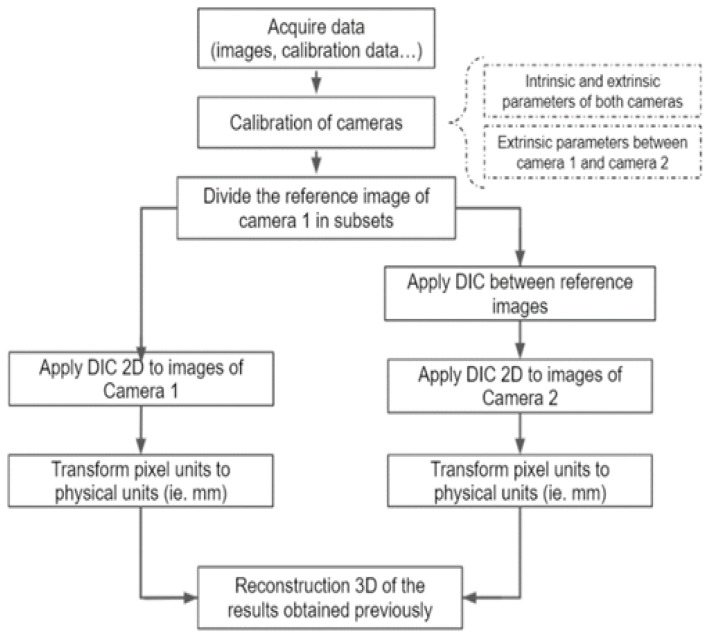
Structural characterization with the projected 3D-DIC configuration (schematic illustration).

Figure 3 describes the developed procedure for structural characterization using projected 3D-DIC. First, before obtaining the calibration images, the high-speed sensors, the optical channel for speckle pattern projection and the sample should be perfectly aligned and uniformly illuminated for 3D-DIC applications. The high-precision rotation stage ensures an accurate orientation of the cameras at 30 degrees in relation to the sample for stereo imaging. This configuration resulted in a scale of 0.5 mm/pixel. Once the speckle pattern is focused, the image acquisition can be performed on unloaded and loaded state images. Then, image processing algorithms and routines were developed to obtain the 3D field displacement and include: (i) the calibration and distortion parameters estimation, (ii) the alignment algorithm to obtain the displacement between subsets (2D-DIC) and (iii) the 3D reconstruction.

#### 2.2.1. Camera Calibration Parameters

**Figure 4:**
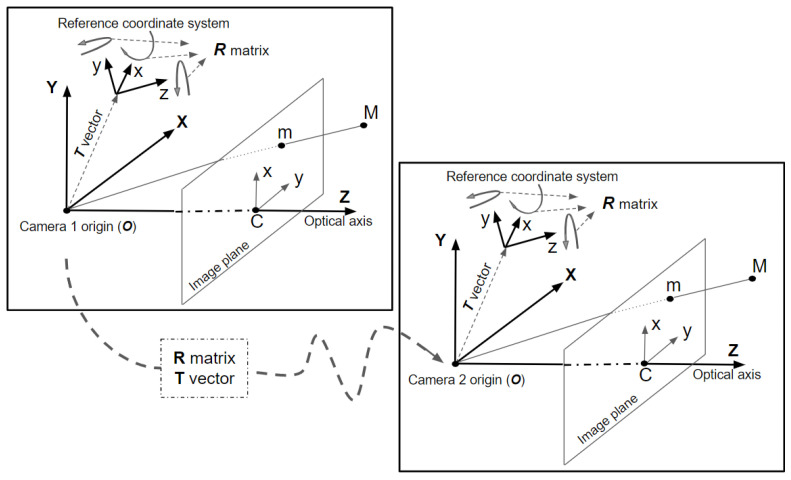
Camera coordinate system composed of the camera systems of both cameras: camera 1 (**left**) and camera 2 (**right**). The relation between both coordinate systems is given by a rotation matrix (R) and a translation vector (T).

The calibration of the high-speed cameras was performed in two different steps: (i) data acquisition for the calibration; and (ii) calculation of the 3D coordinates through an iterative process that requires the estimation of intrinsic (the optical characteristics of the sensor) and extrinsic (position and orientation of the sensor in the set-up) parameters. Figure 4 shows the scheme of the coordinate system. To calibrate a single camera, the method developed by Zhang [49] was implemented, where a set of images of a chessboard with different positions and orientations covering the largest possible area of the analysis region was required. The relationship between 3D points and planar points is defined by Equation (1), using Zhang’s notation.

A 3D point is defined by ${\left[\begin{array}{cccc} X & Y & Z & 1 \end{array}\right]}^{T}$ and the coordinates in the image plane are given by ${\left[\begin{array}{ccc} u & v & 1 \end{array}\right]}^{T}$. The matrix constituted by the intrinsic parameter set is denoted by A. In this matrix, $u_{0}$ and $v_{0}$ are the coordinates of the principal point resulting from the intersection of the image plane with the optical axis, which is represented by C in Figure 4. Coefficients α and β are the focal length and γ is the skew coefficient. The extrinsic parameter set is constituted by rotation matrix R and the translation vector T between the world coordinate system and the camera system. Finally, λ is an arbitrary scale factor.

(1) $$\lambda \left[\begin{array}{c} u\\ v\\ 1 \end{array}\right]=A\left[R|T\right]\left[\begin{array}{c} \begin{array}{c} X\\ Y\\ Z \end{array}\\ 1 \end{array}\right]=\left[\begin{array}{ccc} \alpha & \gamma & u_{0}\\ 0 & \beta & v_{0}\\ 0 & 0 & 1 \end{array}\right]\left[\begin{array}{cc} \begin{array}{cc} r_{1,1} & r_{1,1}\\ r_{1,1} & r_{1,1}\\ r_{1,1} & r_{1,1} \end{array} & \begin{array}{cc} r_{1,1} & r_{1,1}\\ r_{1,1} & r_{1,1}\\ r_{1,1} & r_{1,1} \end{array} \end{array}\right]\begin{array}{c} \begin{array}{c} X\\ Y\\ Z \end{array}\\ 1 \end{array}\;$$

The following items describe the process of solving the single-camera calibration problem:

1. A set of thirty images of the chessboard with different orientations was recorded. The coordinate of each corner was obtained (${\left[\begin{array}{ccc} u & v & 1 \end{array}\right]}^{T}$) and it was associated with the three-dimensional point defined by ${\left[\begin{array}{cccc} X & Y & Z & 1 \end{array}\right]}^{T}$;
2. The analytical solution of Equation (1) was calculated using Zhang’s method;
3. A nonlinear optimization based on the maximum likelihood criterion was developed. The procedure followed is explained in detail in Appendix A.

The two high-speed cameras were synchronized to obtain the images simultaneously for stereoscopic calibration and measurements. Therefore, the rotation matrix R and the translation vector T between the two cameras used for that pairs of calibration images were obtained. Each pair of images correspond with the same chessboard scene. The nonlinear iterative procedure for the stereoscopic calibration is described in Appendix B.

#### 2.2.2. Distortion Correction

Nonlinear distortion is inherent to imaging systems: radial distortion [50] and tangential distortion [51]. The undistorted image coordinates $\left(\begin{array}{cc} \tilde{u} & \tilde{v} \end{array}\right)$ are expressed by Equations (2) and (3), where x and y are the coordinates with respect to the principal point of image plane (C in Figure 4), $k_{i}$ is the coefficient of distortion and r is given by $r=x^{2}+y^{2}$.

(2) $$\tilde{u}=u+x\left[\underset{\mathrm{radial}}{\underset{︸}{k_{1}r^{2}+k_{2}r^{4}+k_{3}r^{6}}}+\underset{\mathrm{tangential}}{\underset{︸}{2k_{4}xy+k_{5}\left(r^{2}+2x^{2}\right)}}\right]$$

(3) $$\tilde{v}=v+y\left[\underset{\mathrm{radial}}{\underset{︸}{k_{1}r^{2}+k_{2}r^{4}+k_{3}r^{6}}}+\underset{\mathrm{tangential}}{\underset{︸}{k_{4}\left(r^{2}+2x^{2}\right)+2k_{5}xy}}\right]$$

Both Equations (2) and (3) have been integrated into the calibration estimation procedure. In particular, in the nonlinear optimization of single calibration and in the nonlinear optimization employed in the stereoscopic calibration.

#### 2.2.3. Alignment Algorithm

The developed routine to obtain the displacement between two subsets is based on the inverse compositional Gauss–Newton algorithm (IC-GN) [15,52], and it consists of the alignment of a template (deformed) image to a target image. For that, the grey-level intensity of each reference subset is correlated to the grey-level intensity of the corresponding template subset. The mathematical expression of the IC-GN algorithm performed on a zero-mean normalized sum of squared difference (ZNSSD) correlation criteria is shown in Equations (4) to (11); in which functions F and G represent the subset of reference images and the subset of deformed images, respectively. The term H corresponds to the hessian calculated by means of Equation (8), variable p is the pre-computed deformation parameter vector, variable ξ designs the local coordinates of pixel point in each subset and W is the warp function given by Equation (9).

**Figure 5:**
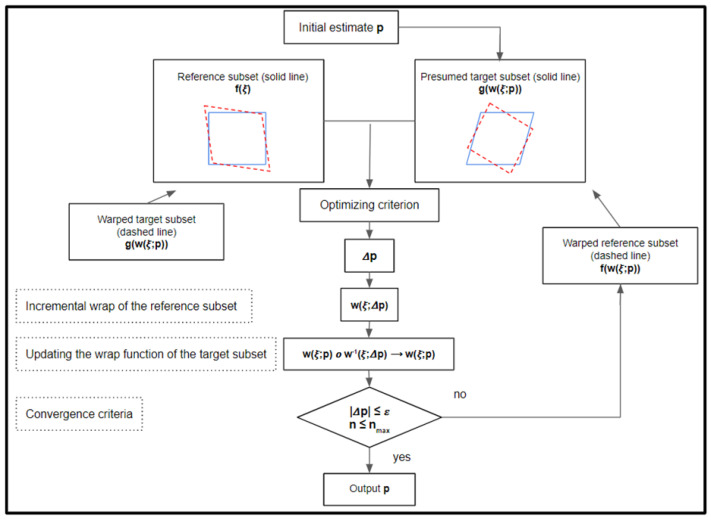
Scheme of the principle of the inverse compositional Gauss–Newton algorithm.

The working principle of the IC-GN algorithm is represented in Figure 5. The initial estimation of $p=\left[\begin{array}{cccccc} u & 0 & 0 & v & 0 & 0 \end{array}\right]$ is usually calculated by means of the direct application of the ZNSSD criteria or another equivalent criteria, such as zero normalized cross-correlation (ZNCC). This allows us to obtain an initial estimation of the u and v components of the displacement vector between the reference subset and the target subset. After that, an iterative loop is performed until achieving the convergence criteria (represented in Figure 5).

(4) $$C_{ZNSSD}\left(\Delta p\right)=\underset{\xi }{\sum }{\left\{\frac{F\left(x+W\left(\xi ;\Delta p\right)\right)-\bar{F}}{\Delta F}-\frac{G\left(x+W\left(\xi ;p\right)\right)-\bar{G}}{\Delta G}\right\}}^{2}\;$$

(5) $$\Delta F=\sqrt{\underset{\xi }{\sum }{\left(F\left(x+W\left(\xi ;\Delta p\right)\right)-\bar{F}\right)}^{2}}$$

(6) $$\Delta G=\sqrt{\underset{\xi }{\sum }{\left(G\left(x+W\left(\xi ;\Delta p\right)\right)-\bar{G}\right)}^{2}}$$

(7) $$\Delta p=-H^{-1}\underset{\xi }{\sum }{\left[\nabla F\frac{\partial W}{\partial p}\right]}^{T}\left[F\left(x+\xi \right)-\bar{F}-\frac{\Delta F}{\Delta G}\left(G\left(x+W\left(x;p\right)\right)-\bar{G}\right)\right]\;$$

(8) $$H=\underset{\xi }{\sum }{\left[\nabla F\frac{\partial W}{\partial p}\right]}^{T}\left[\nabla F\frac{\partial W}{\partial p}\right]$$

(9) $$W\left(\xi ,p\right)=\left[\begin{array}{c} \left(1+u_{x}\right)\Delta x+u_{y}\Delta y+u\\ v_{x}\Delta x+\left(1+v_{y}\right)\Delta y+v\\ 1 \end{array}\right]$$

(10) $$p=\left[u,u_{x},u_{y},v,v_{x},v_{y}\right]$$

(11) ξ=[Δx,Δy,1]

#### 2.2.4. Three-Dimensional Reconstruction

The three-dimensional reconstruction or triangulation is the reconstruction of a 3D point from its 2D projections from two or more cameras. The least square method (LSM) [53] was implemented because of its accuracy and computational efficiency. The relationships between the left image coordinate $\left(\begin{array}{cc} u^{l} & v^{l} \end{array}\right)$, the right coordinate $\left(\begin{array}{cc} u^{r} & v^{r} \end{array}\right)$ and the world coordinate system $\left(\begin{array}{ccc} X & Y & Z \end{array}\right)$ are indicated in Equations (12) and (13), respectively [53]. Integrating both equations, the world coordinate system can be expressed by Equation (14), where b is given by (15) and $M^{+}$ is the left-pseudo inverse ($M^{+}={\left(M^{T}M\right)}^{-1}M^{T}$) of M matrix (16).

(12) $$\left[\begin{array}{c} u^{l}\\ v^{l} \end{array}\right]=\left[\begin{array}{ccc} \alpha^{l} & \gamma^{l} & u_{0}^{l}\\ 0 & \beta^{l} & v_{0}^{l} \end{array}\right]\left[\begin{array}{c} X/Z\\ Y/Z\\ 1 \end{array}\right]$$

(13) $$\left[\begin{array}{c} u^{r}\\ v^{r} \end{array}\right]=\left[\begin{array}{ccc} \alpha^{r} & \gamma^{r} & u_{0}^{r}\\ 0 & \beta^{r} & v_{0}^{r} \end{array}\right]\left[\begin{array}{c} \frac{r_{1,1}X+r_{1,2}Y+r_{1,3}Z+t_{1}}{r_{3,1}X+r_{3,2}Y+r_{3,3}Z+t_{3}}\\ \frac{r_{2,1}X+r_{2,2}Y+r_{2,3}Z+t_{2}}{r_{3,1}X+r_{3,2}Y+r_{3,3}Z+t_{3}}\\ 1 \end{array}\right]$$

(14) $$\left[\begin{array}{c} X\\ Y\\ Z \end{array}\right]=M^{+}b\;$$

(15) $$b=\left[\begin{array}{c} \begin{array}{c} 0\\ 0\\ -\left(t_{3}\alpha^{r}+t_{2}\gamma^{r}+t_{3}\left(u_{0}^{r}-u_{r}\right)\right) \end{array}\\ -\left(t_{2}\beta^{r}+t_{3}\left(v_{0}^{r}-v_{r}\right)\right) \end{array}\right]$$

(16) $$M=\left[\begin{array}{ccc} \alpha^{l} & \gamma^{l} & u_{0}^{l}-u^{l}\\ 0 & \beta^{l} & v_{o}^{l}-v^{l}\\ M_{3,1} & M_{3,2} & M_{3,3}\\ M_{4,1} & M_{4,2} & M_{4,3} \end{array}\right]\;$$

(17) $$M_{3,1}=r_{1,1}^{C}\alpha^{r}+r_{2,1}^{C}\gamma^{r}+r_{3,1}^{C}\left(u_{0}^{r}-u_{r}\right)$$

(18) $$M_{3,2}=r_{1,2}\alpha^{r}+r_{2,2}\gamma^{r}+r_{3,2}\left(u_{0}^{r}-u_{r}\right)$$

(19) $$M_{3,3}=r_{1,3}\alpha^{r}+r_{2,3}\gamma^{r}+r_{3,3}\left(u_{0}^{r}-u_{r}\right)$$

(20) $$M_{4,1}=r_{2,1}\beta^{r}+r_{3,1}\left(v_{0}^{r}-v_{r}\right)\;$$

(21) $$M_{4,2}=r_{2,2}\beta^{r}+r_{3,2}\left(v_{0}^{r}-v_{r}\right)\;$$

(22) $$M_{4,3}=r_{2,3}\beta^{3}+r_{3,3}^{C}\left(v_{0}^{r}-v_{r}\right)$$

### 2.3. Projected 3D-DIC: Test and Validation

**Figure 6:**
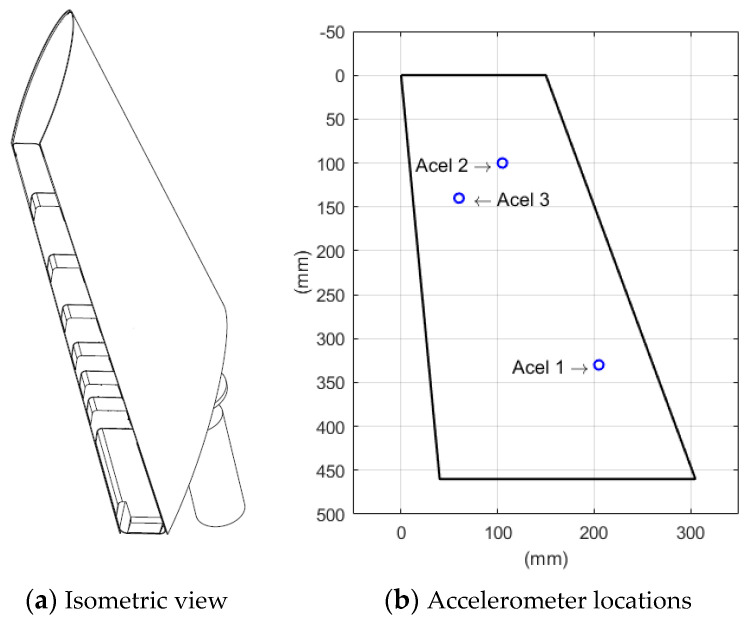
Unmanned Aerial Vehicle (UAV) wing made of composite material: (**a**) Isometric view and (**b**) dimensions and accelerometer locations (Accelerometer 1: Acel 1; Accelerometer 2: Acel 2; Accelerometer 3: Acel 3). Mass of the tested system: 530 g (UAV wing) + 140 g (steel adaptor) + 25.8 g (3 accelerometers).

The structure under testing was the horizontal tail plain (HTP) of the DIANA Unmanned Aerial Vehicle (UAV) with a mass of 530 g and the following dimensions: 455 mm (long) × 265 mm (wide). The DIANA UAV was manufactured with HexPly^®^ M56/35%UD194/IMA12K prepreg, and it was composed of a main skin made of a symmetric laminate [60,–60,0], a longitudinal stringer and reinforcements in the borders made of a symmetric laminate [60,–60,03]. All mechanical joints were made with adhesive. For validation, three accelerometers were placed in the structure (mass of the accelerometer: 8.6 g). Their positions are described in Table 1 (referenced to the upper left vertex) and illustrated in Figure 6. In this study, the type of accelerometer employed was a triaxial sensor with a measuring range of 50 g, a typical sensitivity of 100 Mv/g and an analysis range of up to 10 kHz. For the series of test, the UAV wing was installed on a slipping table fixed to an electromagnetic shaker (LDS 406) powered by a PA100E power amplifier. The drive signal, as well as the acquisition of the accelerometer signals, was performed by means of an LDS Focus II acquisition system. First, a modal identification survey to identify the natural frequencies of the structure was performed; and then the base of the structure was displaced sinusoidally at each natural frequency in order to validate the projected 3D-DIC with the accelerometers.

## 3. Results

In order to evaluate the potential of the projected 3D-DIC for long-duration random vibration measurements, a series of trials were conducted focusing on two major results: (i) modal characterization, which means the modal identification survey to obtain the natural frequencies of the structure inherent to the vibration testing; and (ii) validation of the method with excitation of the UAV wing at the detected natural frequencies of the structure. The 3D-DIC results were compared to the accelerometers’ measurements.

### 3.1. Modal Characterization

**Figure 7:**
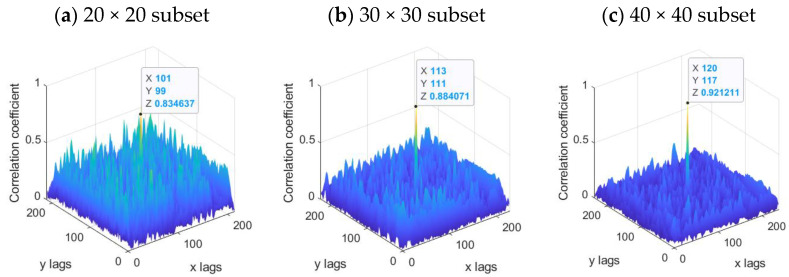
Distribution of the cross-correlation coefficients for three different subset sizes: (**a**) 20 × 20 subset, (**b**) 30 × 30 subset and (**c**) 40 × 40 subset.

For modal determination, a frequency sweep from 0 to 200 Hz was performed to obtain the most relevant natural frequencies of the structure (accelerometers) by programming a sine test at a rate of 1/8 min with a frequency resolution that increases linearly with the frequency sweep. The accelerometers show a resonance frequency of 10 kHz in order to ensure high accuracy in the tested frequency range. The most relevant natural frequencies obtained from the UAV wing were: 42.8 Hz (bending), 57.5 Hz (twisting), 83.1 Hz (twisting) and 97.5 Hz (twisting). Hence, the high-speed cameras were programmed for high-frequency sampling (1000 Hz) at maximum resolution (1024 × 1024 pixels). Three different subset sizes were considered: 20 × 20 pixels, 30 × 30 pixels and 40 × 40 pixels. Figure 7 shows the cross-correlation coefficients for each subset size. It should be noted that as the size of the subset decreases, the probability that the cross-correlation provides erroneous displacements increases mainly due to two reasons: (i) decreasing the subset size increases the magnitude of the residual correlation coefficients, which increases the probability of obtaining wrong displacements; (ii) the smaller the size of the subset, the greater the probability that any of the subsets is empty, with a low number of speckle points in its interior. In the case of applying the cross-correlation to an empty subset, the displacement obtained would be a random number. For the subset size of 20 × 20 pixels, the cross-correlation residues reached values comparable to the maximum value of cross-correlation coefficients. The subset sizes of 30 × 30 and 40 × 40 pixels showed reliable results, so for further analysis, the configuration with the smaller subset size (30 × 30 pixels) was considered.

**Figure 8:**
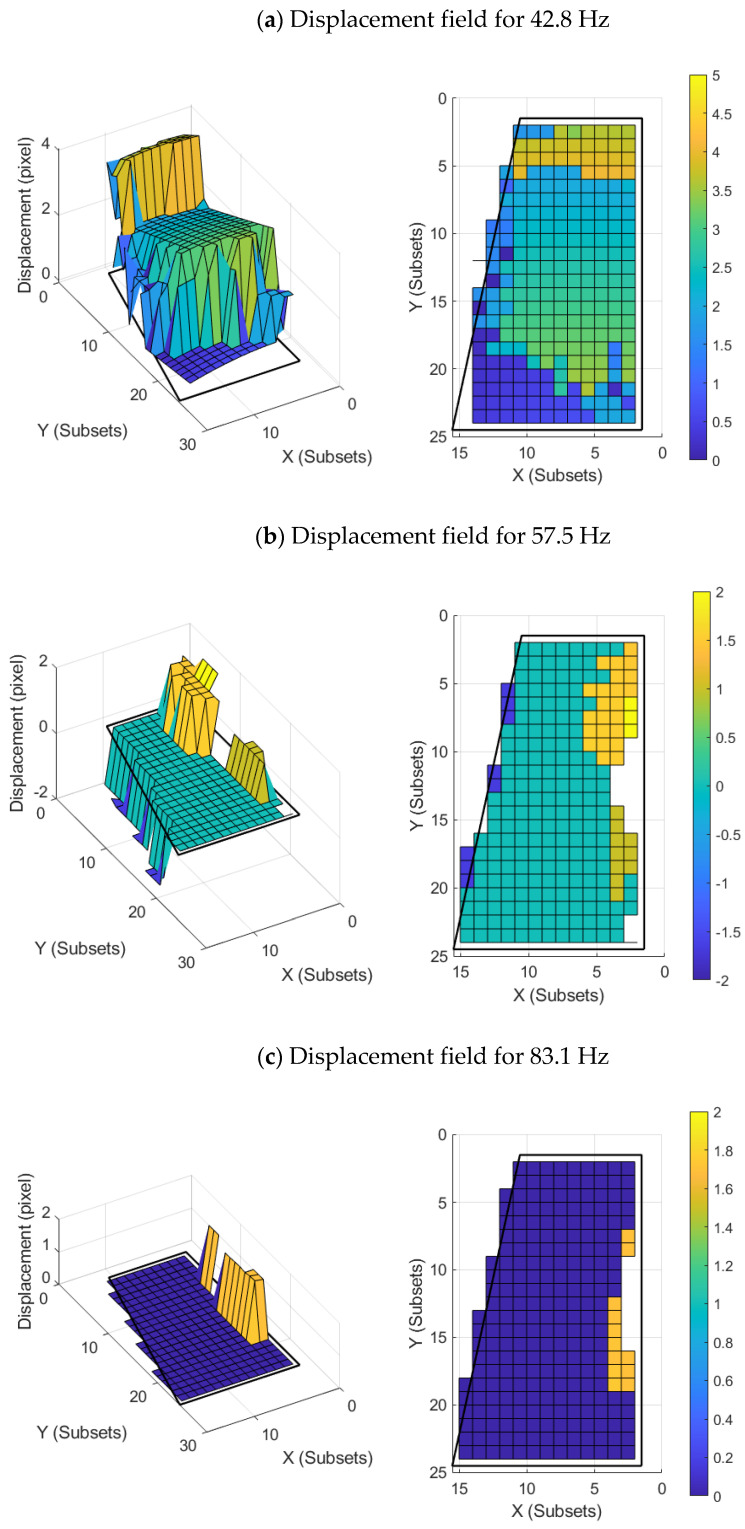

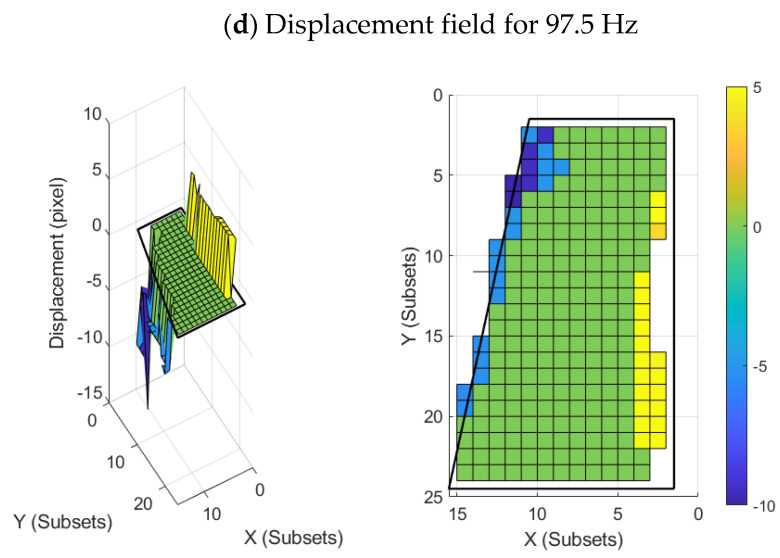
Static representations of the vibration mode for the first relevant natural frequencies from two different perspective points: (**a**) displacement field for 42.8 Hz, (**b**) displacement field for 57.5 Hz, (**c**) displacement field for 83.1Hz and (**d**) displacement field for 97.5 Hz. The color bars represent the displacement field (pixels).

**Figure 9:**
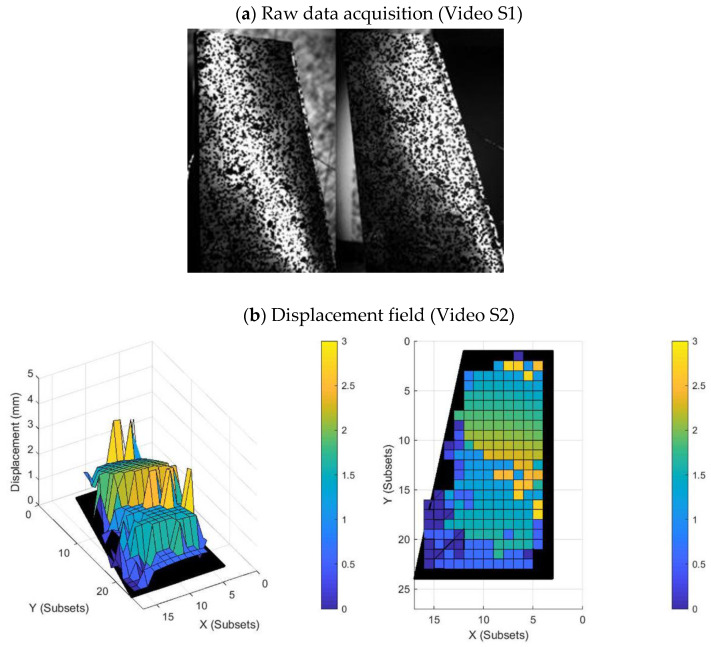
Screenshots of supplementary videos: (**a**) raw data acquisition (Video S1) and (**b**) the displacement field corresponding to the post-processing of raw data from two different perspective points (Video S2). The color bars represent the displacement field (mm).

Figure 8 shows the amplitude of the displacements for each natural frequency. It should be mentioned that the UAV wing was excited to each of these natural frequencies by means of a sine load of the natural frequency. The amplitude of the displacement was calculated using expression (23):

(23) $$D=\sqrt{d_{X}^{2}+d_{Y}^{2}+d_{Z}^{2}}$$

where $d_{X}$, $d_{Y}$ and $d_{Z}$ represent the displacement of each component and D represents the total displacement. Moreover, the sign of displacement was taken into account. The parts of the wing with positive displacement represent an area of the UAV wing that has deformed in the opposite direction to areas with negative displacement. The parts of the wing with null displacement represent a wing area that has not been deformed. Figure 8, Videos S1 and S2 show the raw data (unprocessed images of camera 1 and camera 2) and the temporal evolution of the displacements for the first natural frequency (Figure 9 shows a screenshot of each visualization, the videos can be found as supplementary material).

### 3.2. Experimental Validation

**Figure 10:**
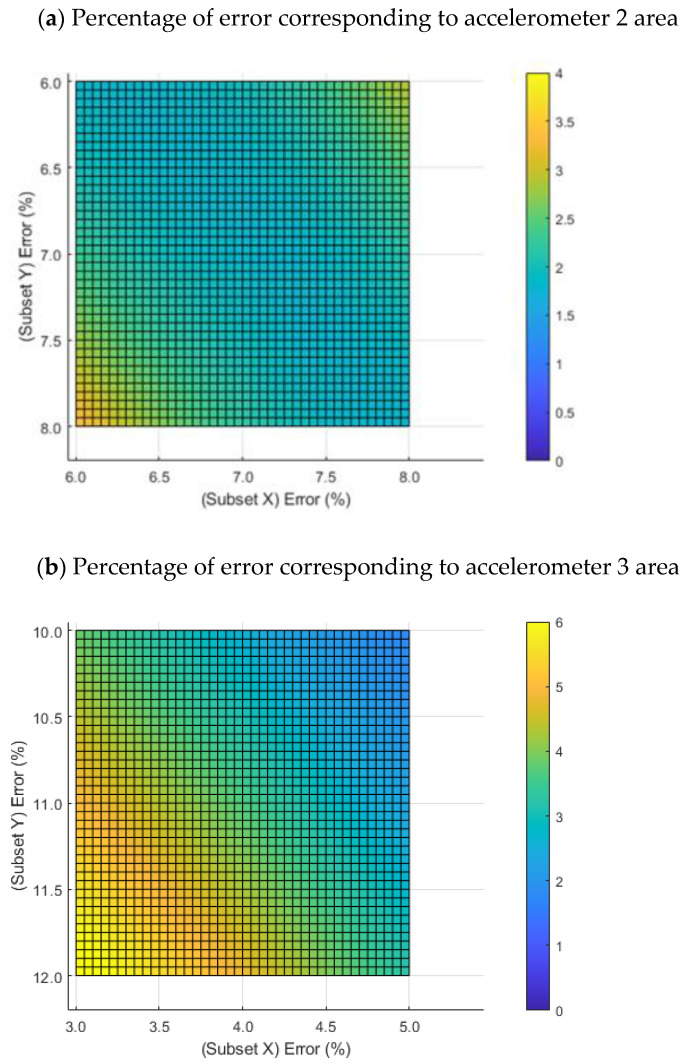
Percentage of error as a result of contrasting the ratio of the accelerometers with respect to the displacements calculated by the 3D-DIC technique in a surface range of −1 to +1 subsets around the accelerometer: (**a**) accelerometer 2; (**b**) accelerometer 3. The color bars represent the percentage of error.

The displacements obtained with the projected 3D-DIC for the first vibration mode (42.8 Hz) were compared with the results obtained with the accelerometers. The values of the accelerometers were normalized with respect to accelerometer 1, the ratio between accelerometers 2 and 1 was 2.75 and the ratio between accelerometers 3 and 1 was 3.25. The displacements obtained with the projected 3D-DIC were also normalized with respect to displacement corresponding to coordinates of accelerometer 1. For that, an offset is applied to all displacements in order to fix the displacement of accelerometer 1 to 1 pixel. After that, the displacements were contrasted with respect to the ratios of accelerometers 2 and 3. Assuming that the accelerometers were not perfectly placed in the coordinates indicated in Table 1, a sweep was performed from −1 subset to +1 subset around the position of both accelerometers (accelerometers 2 and 3). The result of this operation is shown in Figure 10. For Figure 10a, the errors ranged from 1.8% (center) to 3.38 % (bottom left), while for Figure 10b, the error ranged from 3.7% (center) to 6.3% (bottom left).

## 4. Discussion

This study proposed an accurate method for full-field vibration measurement. The method is based on projected 3D-DIC and is capable of non-invasively identifying the full-field mode shapes and natural frequencies of a UAV wing made of composite material, which could potentially benefit structural dynamics and health monitoring applications [54]. Previous studies described fringe patterns [55,56], laser speckle [41,57] or a combination of both [40] to create a non-invasive pattern for full-field vibration measurement in DIC applications. In a recent study, Felipe-Sesé et al. [40] proposed a combination of a fringe pattern, a laser speckle and a single-camera DIC to measure the three-dimensional shapes and the associated field of displacements of a plate excited by a shaker. The authors validated their results with theoretical predictions and obtained a maximum error near the edges of the structure of 4.95%. Moreover, Pang et al. [58], evaluated the flexural behavior of concrete sleepers with a laser speckle imaging sensor. The authors validated the methodology with foil strain gauges and described a maximum error of 7.15% in their measurements.

In this study, a speckle pattern projection has been chosen not only as an alternative to the conventional invasive painted speckle patterns but also to other non-invasive and complex options (i.e., laser speckle or fringe patterns). Overall, the study holds similarities with those recently reported in the state-of-the-art and includes the novelty of the analysis on a composite material at different vibration modes, showing high sensitivity to detect the temporal evolution of the vibration modes. Accurate 3D mode shapes were obtained for different resonances, the results show a percentage of error below 3% and 5% compared with reference accelerometers 2 and 3, respectively. Accelerometer 3 was placed near the edge of the structure, and the discontinuity at this region might be the reason for a reduced accuracy [59,60].

Although the measurements were performed for a sampling frequency of 1000 Hz, one of the limitations of the study is that the more relevant vibration modes of the UAV structure occurred in the range from 0 to 100 Hz. Another limitation is associated with the out-of-plane motion tracking since the projected speckle pattern does not move tightly together with the surface of the structure. However, the latter might not be considered a weakness for vibrational analysis because the excitation of the structure is applied longitudinally on each axis, and the whole out-of-plane displacement measurements of the measured area are fundamental for the evaluation of the vibration modes of the structure. The advantages of the proposed methodology are the high-frequency capacity (up to 4000 Hz under the same configuration) and great versatility to (i) customize the speckle projected pattern for canceling the residues of the cross-correlation coefficients and (ii) configurate the set-up for measurements in a selected spectrum range (i.e., near-infrared). This methodology could be directly applied for vibrational analysis of optical surfaces, such as solar cells, glass structures and lenses, or even for biomechanical analysis in human tissues (e.g., transparent tissues such as the cornea).

## 5. Conclusions

3D-DIC based on speckle pattern projection is an accurate method to characterize the structural dynamics of a composite material. The proposed method successfully obtained full-field vibration measurement with high spatial resolution and is capable of reliably extracting the shape and intensity, as well as the out-of-plane displacement fields and mode shapes of the structure.

## 6. Patents

The authors indicate the following financial disclosure(s): PCT/ES2018/070757, “Device and method for obtaining mechanical, geometric and dynamic measurements of optical surfaces” (P.P.-M., A.F.L.).

## Figures and Tables

**Table 1:** Coordinates of the accelerometers.

Accelerometers	Coordinates (mm)	Coordinates (Subsets)
Accelerometer 1	[205,330]	[11,21]
Accelerometer 2	[105,100]	[7,7]
Accelerometer 3	[60,140]	[4,11]

**Figure A1:**
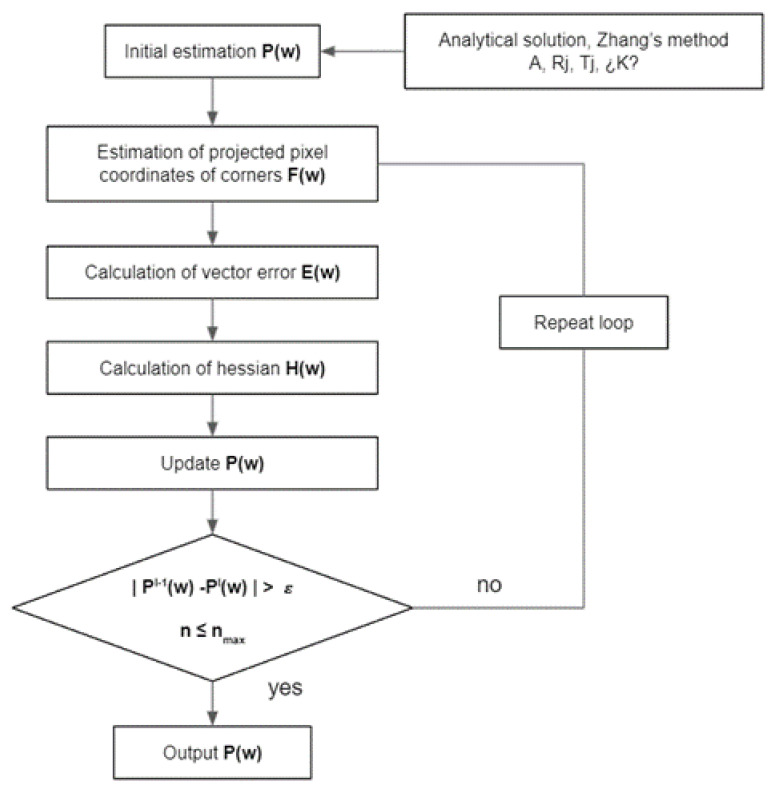
Iterative refinement of single-camera parameters.

**Figure A2:**
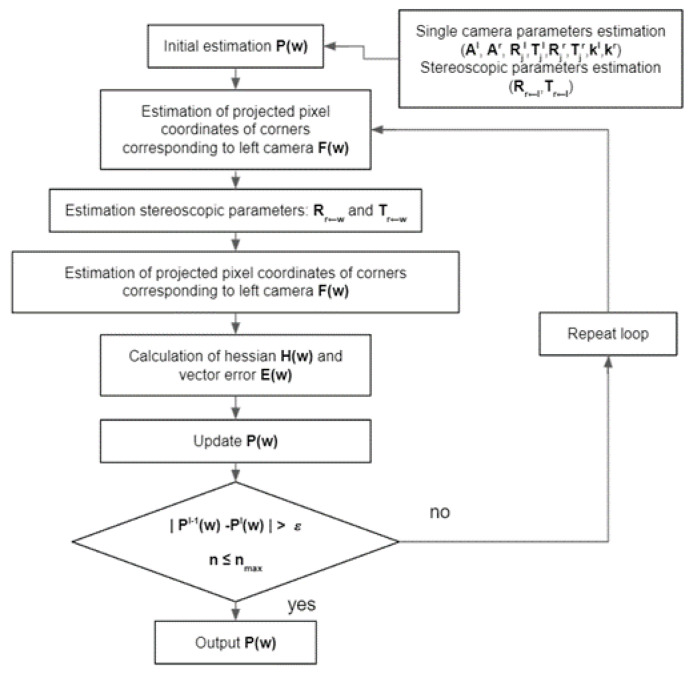
Iterative refinement of stereoscopic parameters.

## Data Availability

Not applicable.

## Supplementary Materials

The following supporting information can be downloaded at: https://www.mdpi.com/article/10.3390/s22249766/s1, Video S1: Raw data visualization, Video S2: Displacement fields.

## Appendix A. Estimation of the Single Camera Calibration Parameters

After taking the images of the chessboard calibration pattern, all the coordinates of the points through Harris corner detection were solved [61]. Then, the camera calibration parameters were calculated by minimizing the difference between the two-dimensional coordinates at the edges of the calibration test by means of Equation (1). The procedure to obtain the solution was explained by Zhang [49]; however, it should be noted that the analytical solution of Zhang’s method needs further refinement because of nonlinear distortion. Therefore, in order to minimize the error of the calibration parameters, a nonlinear iterative loop was implemented by using the following equations. Figure A1 illustrates the method for the estimation of the single-camera calibration parameters.

(A1) $$P{\left(w\right)}^{l+1}=P{\left(w\right)}^{l}+\left\{1-{\left(1-\eta \right)}^{l+1}\right\}H{\left(w\right)}^{-1}E\left(w\right)$$

(A2) $$\rho =\left\{A,k,R_{j},T_{j}\right\}$$

(A3) $$w=\left\{\rho ,M_{i,j}\right\}$$

(A4) $$F\left(w\right)=\hat{m}\left(w\right)=\left[\begin{array}{c} x_{p}\\ y_{p} \end{array}\right]=\left[\begin{array}{c} \alpha x_{u}+\gamma y_{u}+u_{0}\\ \beta y_{u}+v_{0} \end{array}\right]\;$$

(A5) $$x_{u}=\frac{X_{c}}{Z_{c}}a+2k_{4}\frac{X_{c}}{Z_{c}}\frac{Y_{c}}{Z_{c}}+k_{5}\left(r^{2}+2\frac{X_{c}^{2}}{Z_{c}^{2}}\right)$$

(A6) $$y_{u}=\frac{Y_{c}}{Z_{c}}a+k_{4}\left(r^{2}+2\frac{Y_{c}^{2}}{Z_{c}^{2}}\right)+2k_{5}\frac{X_{c}}{Z_{c}}\frac{Y_{c}}{Z_{c}}$$

(A7) $$a=1+k_{1}r^{2}+k_{2}r^{4}+k_{3}r^{6}$$

(A8) $$\left[\begin{array}{c} X_{c}\\ Y_{c}\\ Z_{c} \end{array}\right]=R\left[\begin{array}{c} X\\ Y\\ Z \end{array}\right]+T\;$$

(A9) $$E\left(w\right)=m_{i,j}-\hat{m}\left(w\right)$$

(A10) $$H\left(w\right)=\left[\begin{array}{ccc} \begin{array}{cc} \frac{\partial F\left(w\right)}{\partial w_{1}\partial w_{1}} & \frac{\partial F\left(w\right)}{\partial w_{1}\partial w_{2}}\\ \frac{\partial F\left(w\right)}{\partial w_{2}\partial w_{1}} & \frac{\partial F\left(w\right)}{\partial w_{2}\partial w_{2}} \end{array} & \cdots & \begin{array}{c} \frac{\partial F\left(w\right)}{\partial w_{1}\partial w_{k}}\\ \frac{\partial F\left(w\right)}{\partial w_{2}\partial w_{k}} \end{array}\\ \vdots & \ddots & \vdots \\ \begin{array}{cc} \frac{\partial F\left(w\right)}{\partial w_{k}\partial w_{1}} & \frac{\partial F\left(w\right)}{\partial w_{k}\partial w_{2}} \end{array} & \cdots & \frac{\partial F\left(w\right)}{\partial w_{k}\partial w_{k}} \end{array}\right]$$

where,

- A is the matrix of the intrinsic parameters constituted by α, β, γ, $u_{0}$ and $v_{0}$ according to Zhang’s nomenclature [49].
- k represents a vector with radial and tangential distortion parameters.
- $R_{j}$ is the rotation matrix, which corresponds to image j.
- $T_{j}$ is the translation vector, which corresponds to image j.
- $P{\left(w\right)}^{l}$ is the vector constituted by w parameters, which corresponds to iteration l.
- E(w) is a vector with the error estimation of each corner.
- $H{\left(w\right)}^{-1}$ is the inverse matrix of the hessian matrix defined in Equation (A10).
- η is the smoothing factor.
- $m_{i,j}$ is the two-dimensional corner coordinates of the chessboard, they must be obtained with Harris corner detector.
- $\hat{m}\left(w\right)$ are the estimated corner coordinates by Equation (A4).
- $X_{c}$, $Y_{c}$ and $Z_{c}$ are three-dimensional coordinated in the camera plane.
- $x_{u}$ and $y_{u}$ are undistorted coordinates.
- $x_{p}$ and $y_{p}$ are the projected pixel coordinates of corners.
- $M_{i,j}$ is the three-dimensional corners’ coordinates.

## Appendix B

The stereoscopic calibration parameters are constituted by the rotation matrix from the left camera system to the right camera system ($R_{r\leftarrow l}$), which defines the orientation between both cameras and the translation vector between the left camera system to the right camera system ($T_{r\leftarrow l}$). Just as the estimation of the single camera parameters, the solution was calculated by minimizing the difference between the two-dimensional known coordinates (Harris corner detector) and the two-dimensional estimated coordinates of the corners. However, it is important to highlight that before estimating the stereoscopic parameters, the parameters of each camera should be calculated. As shown in Equation (A11), the number of parameters has grown significantly by incorporating the left single-camera parameters (denoted with superscript l), the right single-camera parameters (defined with superscript r) and the estereoscopic parameters (without superscript). The iterative refinement starts with an initial estimation of ρ parameters. The majority of the parameters were previously calculated by means of single-camera calibration and the initial estimation of $R_{r\leftarrow l}$ and $T_{r\leftarrow l}$ were calculated according to the mean values obtained via Equations (A13) and (A14).

(A11) $$\rho =\left\{A^{l},A^{r},k^{l},k^{r},R_{j}^{r},R_{j}^{l},R_{r\leftarrow l},T_{j}^{r},T_{j}^{l},T_{r\leftarrow l}\right\}$$

(A12) $$w=\left\{\rho ,M_{i,j}^{r},M_{i,j}^{l}\right\}$$

(A13) $$R_{r\leftarrow w}=R_{r\leftarrow l}R_{l\leftarrow w}$$

(A14) $$T_{r\leftarrow w}=T_{r\leftarrow l}+R_{r\leftarrow l}T_{l\leftarrow w}$$

The iterative loop (see Figure A2) was defined by the following steps:

- Estimation of the projected pixel coordinates corresponding to the corners of the images (left camera) by using Equation (A4).
- Estimation of the rotation matrix from the world system to the right camera system ($R_{r\leftarrow w}$) by using Equation (A13) and the translation vector from the world system to the right camera system ($T_{r\leftarrow l}$) by means of Equation (A14).
- Estimation of the projected pixel coordinates corresponding to the corners of the images (right camera) by using Equation (A4).
- Calculation of the vector error as the difference between the two-dimensional known coordinates and the estimated coordinates of the corners of all the images (both cameras, left and right).
- Extraction of the hessian matrix by Equation (A10).
- Update of the parameters by means of Equation (A1).
